# A strategy to determine operating parameters in tissue engineering hollow fiber bioreactors

**DOI:** 10.1002/bit.23062

**Published:** 2011-06

**Authors:** RJ Shipley, AJ Davidson, K Chan, JB Chaudhuri, SL Waters, MJ Ellis

**Affiliations:** 1 Oxford Centre for Industrial and Applied Mathematics, Mathematical Institute24–29 St. Giles', Oxford OX1 3LB, UK; telephone: 01865270744; fax: 01865270515.; 2 Centre for Regenerative Medicine, Department of Chemical Engineering, University of BathBath, UK; 3 Centre for Mathematical Biology, Mathematical InstituteOxford, UK

**Keywords:** tissue engineering, bioreactor, oxygen, mass transport, mathematical modeling

## Abstract

The development of tissue engineering hollow fiber bioreactors (HFB) requires the optimal design of the geometry and operation parameters of the system. This article provides a strategy for specifying operating conditions for the system based on mathematical models of oxygen delivery to the cell population. Analytical and numerical solutions of these models are developed based on Michaelis–Menten kinetics. Depending on the minimum oxygen concentration required to culture a functional cell population, together with the oxygen uptake kinetics, the strategy dictates the model needed to describe mass transport so that the operating conditions can be defined. If *c*_min_ ≫ *K*_m_ we capture oxygen uptake using zero-order kinetics and proceed analytically. This enables operating equations to be developed that allow the user to choose the medium flow rate, lumen length, and ECS depth to provide a prescribed value of *c*_min_. When 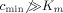, we use numerical techniques to solve full Michaelis–Menten kinetics and present operating data for the bioreactor. The strategy presented utilizes both analytical and numerical approaches and can be applied to any cell type with known oxygen transport properties and uptake kinetics.

## Introduction

Hollow fiber bioreactors (HFBs) are ideal for tissue engineering on a clinical scale because the large surface area to volume ratio will reduce the requirements of reagents, labor, and space: a hollow fiber system can be used to culture the same number of cells in 0.58 L as 1 m^3^ using standard flask culture techniques (Ellis et al., 10), and large cell numbers of up to 2 × 10^8^ cell/mL can be obtained (Scragg, 31). Knazek et al. (18) were the first to report using a HFB for mammalian cell culture; since then the use of HFBs for mammalian cell expansion has become well documented (Tharakan and Chau, 37) and several cell types have been cultured in HFBs including lymphocytes (Gramer and Poeschl, 13; Gloeckner and Lemke, 12), hepatocytes (Nyberg et al., 23), and the osteogenic cell line 560pZIPv.neo (Ellis and Chaudhuri, 9). There is extensive understanding of HFB fluid dynamics and mass transport obtained from experimental and modeling studies, and a wealth of data on tissue physiology and cell metabolism in vivo and in vitro. For example, Abdullah et al. (2) and Abdullah and Das (1) have focused on high-density bone cell populations, whereas hepatocyte culture has provided a focus for bioartificial liver development through studies such as Hay et al. (14), Kawazoe et al. (16), Nyberg et al. (24), Patzer (27), Sielaff et al. (33), Sullivan et al. (35), and Wurm et al. (40). Together these studies provide insight into the interaction between the cell environment and the fluid dynamics and mass transfer of nutrients across the membrane. Oxygen is recognized as the limiting nutrient with respect to growth of a cell population and has been the most widely modeled (although glucose has also been considered). The uptake of oxygen is usually modeled using Michaelis–Menten kinetics, which captures the dependence on the uptake rate on the underlying concentration.

As a consequence of the nonlinear nature of Michaelis–Menten kinetics, numerical solutions to the transport equations associated with HFBs are commonly seen in literature. These use full Michaelis–Menten; examples of finite difference methods include Pillarella and Zydney (28), whereas examples of finite element methods include Abdullah and Das (1), Chen and Palmer (6), Das (8), Sullivan et al. (35, 36), and Ye et al. (41). Analytical approaches have also been used in literature for situations where Michaelis–Menten can be approximated by zero- or first-order kinetics. Example of zero-order kinetics are Piret and Cooney (29), whereas examples of first-order kinetics are Jayaraman (15) and Kim and Cooney (17). Although Kim and Cooney (17) use first-order kinetics, the functional forms for the substrate concentrations that they determine are not dissimilar to those presented in this article. A good review of a range of transport models in HFBs is given by Brotherton and Chau (4).

To ensure the efficacy of HFB for clinical applications it is necessary to have information that allows accurate and correct operation of the HFB. This article presents a tool to select the modeling approach best suited to obtain cell type-specific operating data. As such, the approach presented here differs significantly from existing studies in the literature. First of all, previous studies have considered only analytical or numerical solutions in isolation. Here we use both approaches, and specify how to differentiate between the two based on cell data. Secondly, the analytical solutions that we present are based on zero-order kinetics and have not been reported previously in the literature. Finally, a strategy is outlined for providing operating data (specifically the lumen length, extra-capillary space (ECS) depth, and lumen flow rate) that ensure the oxygen concentration throughout a HFB is held above a prescribed tissue-specific minimum. When an analytical approach is applicable this data takes the form of operating equations that relate the underlying parameters; for the numerical approach operating data are presented graphically. This strategy enables a user to fix the geometry (e.g., lumen length, ECS depth) and operating conditions (e.g., lumen length) of the bioreactor to obtain their required cell culture environment.

## Theory

### Setup

**Figure 1:**
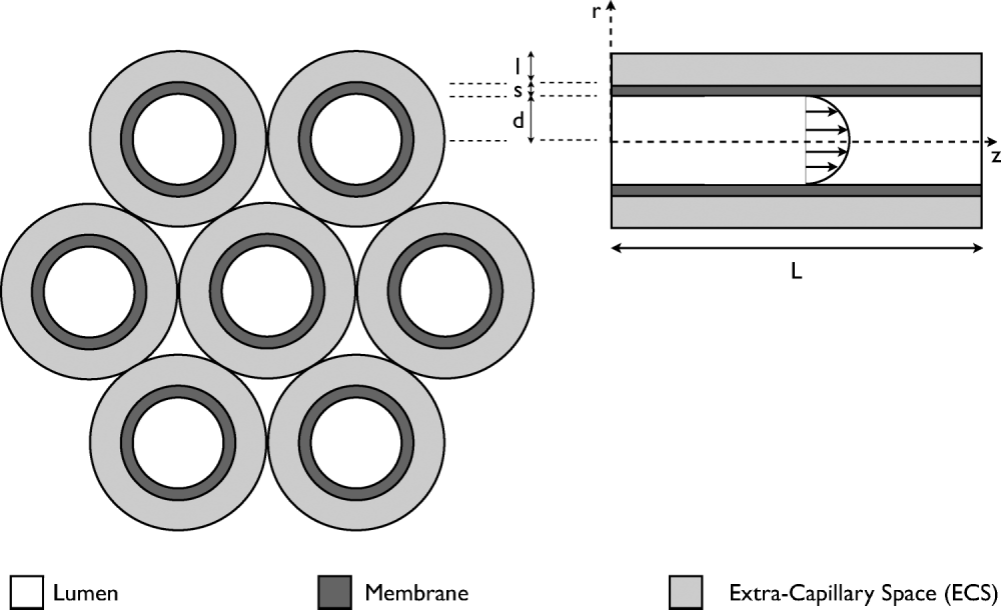
A schematic of the HFB setup. The left-hand schematic shows the structure of a fiber bundle, comprising seven Krogh cylinder units. The right-hand schematic shows a cross-section through an individual fiber, including the fluid velocity profile in the lumen.

The fibers in a HFB fiber bundle are assumed to be Krogh cylinders, so that each fiber is identical and surrounded by an annulus of ECS containing a homogeneous distribution of cells (Krogh, 19). The interstitial space between the Krogh cylinders is neglected as a modeling assumption. In this study, we consider transport in a single Krogh cylinder unit of a HFB bundle. This unit consists of a central lumen with a synthetic porous wall (referred to as the membrane), and surrounding ECS containing cells. Let *z* be the axial direction down the lumen centerline, starting at the lumen inlet (*z* = 0) with the lumen outlet denoted by *z* = *L*. We denote the radius of the lumen by *d*, the depth of the membrane by *s* and the depth of the ECS by *l*. Typical values are *L* = 10 cm, *d* = 100 µm, *s* = 20 µm, and *l* = 600 µm (Ye et al., 42), although these should be varied as part of the bioreactor design process. A schematic of the setup is given in Figure 1.

Culture medium is pumped through the lumen at an imposed flowrate. There is no flow through the inlet to the membrane or ECS, so that fluid enters the system through the lumen only. Although this medium includes a mixture of solutes and proteins, we consider the transport of oxygen alone in this article. This is a widely adopted approach in the literature as oxygen is generally considered to be the rate-limiting nutrient, and reduces the complexity of the modeling process (Martin and Vermette, 22; Piret and Cooney, 29). Oxygen is transported by both advection (by the fluid) and diffusion in the lumen. Furthermore, oxygen diffuses through the membrane and ECS, where it is taken up by the cell population. In the analysis that follows we assume that the cell population is homogeneously distributed throughout the ECS, and neglect expansion of the cell population so that the parameters describing oxygen uptake are constant in time.

Fluid flow in the lumen is described by Poiseuille's law whereas flow in the membrane and ECS is neglected (this is a common modeling assumption for small aspect ratio HFB when there is not a significant pressure drop across the membrane or ECS (Brotherton and Chau, 4; Piret and Cooney, 29)). We denote this fluid velocity in the lumen by 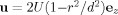, where *U* is the mean velocity (ms^−1^), *r* is the radial coordinate, and **e**_*z*_ is the unit vector in the *z*-direction. The oxygen concentration and flux are denoted by *c* (mol m^−3^) and **J** (mol m^−2^ s^−1^), respectively, with subscripts *l*, *m*, and *e* denoting the values in the lumen, membrane, and ECS, respectively. The oxygen fluxes are

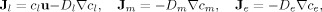
1 where *D*_*l*_, *D*_*m*_, and *D*_*e*_ are the diffusion coefficients for oxygen in the lumen, wall, and ECS, respectively (all assumed constant, with units m^2^ s^−1^). The lumen oxygen flux is comprised of advection due to the fluid velocity, together with diffusion; the membrane and ECS fluxes are comprised of diffusion only. The conservation equations for the concentration of oxygen in each of the regions are:

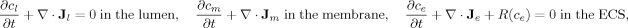
2 where the reaction term *R*(*c*_*e*_) captures the uptake of oxygen by the cells. We will assume Michaelis–Menten kinetics for this reaction term, so that

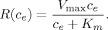
3

It is necessary to prescribe boundary conditions on the internal and external boundaries of the bioreactor. On the lumen/membrane and membrane/ECS boundaries we prescribe continuity of concentration and flux, so that

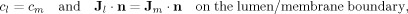
4

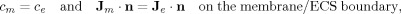
5 where **n** is the unit outward pointing normal to the relevant surface. Finally we prescribe the oxygen concentration as *c*_in_ (mol m^−3^) at the lumen inlet (where *c*_in_ may be chosen to suit the application under consideration), and impose no flux of concentration out of the outer ECS boundary,

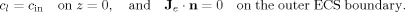
6

The assumption of no flux out of the outer boundary is analogous to a symmetry condition representation of a bundle of fibers. It compares directly to the Krogh cylinder approach used frequently in the literature.

Next the solution of the model (2)–(6) is considered using numerical or analytical techniques. For both strategies a steady-state solution is sought and it is assumed that a 2D axisymmetric geometry is described by the radial coordinate 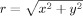 and the axial coordinate *z*.

### Analytical Approach

To pursue an analytical approach, the system of equations given by (2)–(6) can be simplified with various assumptions. First of all the small aspect ratio of a fiber is exploited, defined by 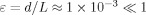. It should be noted that whilst the lumen radius, *d* and fiber length, *L* can both be varied as part of the design process so that neither *d* nor *L* are fixed, *ε* ≪ 1 will be maintained throughout.

It is not possible to make progress analytically using the nonlinear Michaelis–Menten reaction term given by (3). Therefore, we assume that *c*_*e*_ ≫ *K*_*m*_ so that the reaction term *R*(*c*_*e*_) can be approximated by *V*_max_. This is an important assumption and means that predictions of the analytical model are only valid when the ECS oxygen concentration is much larger than the half-maximal oxygen concentration. As such, for cell types where the demand for oxygen is similar to, or smaller than, *K*_*m*_ it will not be appropriate to use the analytical model (in this scenario a numerical approach should be used, as outlined later in the article).

Finally the relative importance of advection and diffusion in the lumen is evaluated by considering the Péclet number, *Pe* = *UL*/*D*_*l*_. In fact it is the reduced Péclet number, 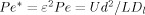, that is critical for this system, as it also takes account of the small aspect ratio of the lumen (it is analogous to the *reduced Reynolds number* that was used to characterize fluid transport for a similar study in Shipley et al. (32)). A large reduced Péclet number indicates an advection-dominated regime, whereas a small reduced Péclet number indicate a diffusion-dominated regime. Typically for this system *U* ≍ 1 cm s^−1^, *L* ≍ 10 cm, and *D* ≍ 10^−9^ m^2^ s^−1^, giving Pe^*^ ≍ 1 so that advection and diffusion are both important in the lumen. It is assumed that Pe^*^ = ε^2^*Pe* is of order 1 in the analysis that follows. For the mathematical detail of the reduction of (2)–(6) based on the assumptions above, together with the solution of the resulting model, please refer to the Supplementary Material A.

The outer radius of the lumen, membrane and ECS (each measured from the lumen centerline) are denoted by *R*_*l*_, *R*_*w*_, and *R*_*e*_ so that *R*_*l*_ = *d*, *R*_*m*_ = *d* + *s*, and *R*_*e*_ = *d* + *s* + *l*. The following dimensionless parameters are also defined:

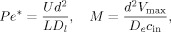
7 which capture the key physical features of the system. As described above, Pe^*^ is the reduced Péclet number and is assumed to be of order 1. The parameter *M* represents the balance of oxygen consumption versus diffusion in the ECS, and can take a range of values depending on the relative importance of these effects.

The analysis described above and in the Supplementary Material results in the following expressions for the oxygen concentration throughout the module:

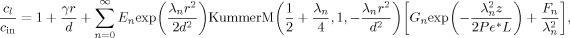
8

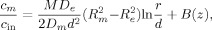
9

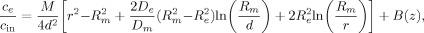
10 where

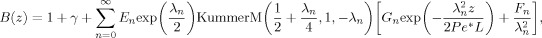
11 and

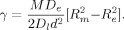
12

Here “KummerM 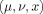
” is the confluent hypergeometric function and is a solution of a specific differential equation, as described in the Supplementary Material A (and discussed in Abramowitz and Stegun, 3). Further *λ*_*n*_, *E*_*n*_, *F*_*n*_, and *G*_*n*_ for 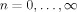 are constants. The *λ*_*n*_ and *E*_*n*_ are the eigenvalues and normalization constants for the Sturm–Liouville problem associated with the system (2)–(6); these are constants independent of the geometry or cell population properties and are provided in the Supplementary Material B for 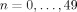. By contrast *F*_*n*_ and *G*_*n*_ are coefficients in a Sturm–Liouville expansion of two different functions, and depend explicitly on the cell population properties (specifically the consumption rate of oxygen) and the geometry of the bioreactor (specifically the radius of the lumen and depths of the membrane and ECS).

Although Equations (8)–(10) appear complex, the behavior that they describe is relatively straightforward to understand: the oxygen concentration in the lumen, membrane, and ECS depends on the radial distance from the lumen centerline. Each solution is also dependent on the distance down the lumen centerline, *z*, as a consequence of advection in the lumen. This is transmitted into the membrane and ECS regions through the function *B*(*z*), which is the lumen concentration value on the lumen wall (i.e., the solution in (8) when *r* = *R*_*l*_ = *d*). This function *B*(*z*) reveals that the concentration decays exponentially down the lumen from a maximum value at the inlet *z* = 0. The remaining terms in the solution for *c*_*m*_ and *c*_*e*_ in (9)–10 describe the radial decay of the oxygen concentration from the outer surface of the membrane as a consequence of oxygen uptake by the cells in the ECS.

Through cell-specific design criteria, we must design the bioreactor to ensure that the oxygen concentration exceeds a prescribed minimum throughout the bioreactor. This minimum oxygen concentration will be achieved at the furthest distance from the inlet, that is, when *r* = *R*_*e*_ and *z* = *L*. Denoting this minimum value by *c*_min_, the analytical method gives the following expression for *c*_min_, in terms of experimentally controlled and cell-specific parameters:

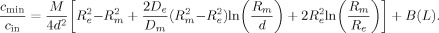
13

### Numerical Approach

For the analytical approach, the full system given by (2)–(6) is solved using finite element method package “COMSOL Multiphysics 3.5a”^1^ to evaluate the dependence of the oxygen concentration on the underlying parameters. The numerical approach is valid for all concentration values; however, the full system of equations must be solved iteratively each time. This is a computationally intensive process and does not provide operating equations that describe the dependence of the minimum oxygen concentration on the underlying parameters. Therefore, the numerical approach will be used when the analytical approach is not valid, that is, when 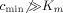. The mesh used for the results in this article consists of approximately 7,000 finite elements (and refining the mesh to 29,312 elements did not change the results to three significant figures).

## Results and Discussion

The analytical and numerical methodologies outlined in the Theory Section will be used to outline a strategy for developing cell-specific operating criteria for the bioreactor. These criteria will then be tested for specific cell types.

### Strategy for Developing Optimal Operating Conditions

To develop operating conditions, it is necessary to understand how the minimum oxygen concentration depends on the geometrical properties of the bioreactor, together with the parameters that can be controlled experimentally. Through this understanding, the HFB can be designed to optimally grow cells of a particular type.

Once a cell type and seeding density are chosen the following parameters are fixed:

1. The maximal oxygen consumption rate, *V*_max_.
2. The half-maximal oxygen concentration, *K*_*m*_.
3. The diffusivity of oxygen in the ECS, *D*_*e*_.

The diffusivity of oxygen in the lumen and membrane (*D*_*l*_ and *D*_*m*_, respectively) are known from the literature or experiments. The outer radii of the lumen and membrane (*R*_*l*_ and *R*_*m*_, respectively) are fixed, and there are specific values of the lumen inlet concentration *c*_in_ and minimum oxygen concentration *c*_min_ that must be achieved. So, the bioreactor design parameters that are left to be determined are:

1. The depth of the ECS, *l* (which determines *R*_*e*_).
2. The length of the lumen, *L*.

Finally, the mean inlet flow rate *U* can be controlled by fixing the volumetric flow rate on the pump used to deliver fluid to the bioreactor.

If *c*_min_ ≫ *K*_*m*_ the analytical approach is valid and the results from the Analytical Approach Section can be used to fix *l*, *L*, and *U*; however, if *c*_min_ ≫ *K*_*m*_ the analytical approach is not valid, and the numerical method must instead be used. These two approaches are detailed below.

***c***_**min**_ **≫** ***K_m_*** The operating conditions are specified for the bioreactor using Equation (13) for the minimum oxygen concentration. When the parameters described above are fixed, only *R*_*e*_ and the ratio *U*/*L* (through Pe^*^) can be determined independently using the analytical approach. Two cases will be considered:

1. The outer radius of the ECS, *R*_*e*_ is fixed, and so Pe^*^ can be determined.
2. The ratio *U*/*L* (and therefore Pe^*^) is fixed, and so *R*_*e*_ can be determined.

For the first case it is assumed that the outer radius of the ECS is fixed so that *R*_*e*_ (and thus *γ*, *F*_*n*_, and *G*_*n*_ for 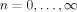) is known. In this case, (13) can be written as the following operating equation for *c*_min_ in terms of the reduced Péclet number Pe^*^:

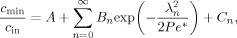
14 where

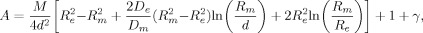
15

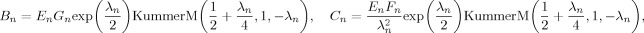
16 are all fixed constants. Given the values of these constants, 14 can be used to determine the value of Pe^*^ (and hence the ratio *U*/*L*) that provides the required value of *c*_min_ (note that it is this ratio rather that the individual values of *U* and *L* that influence the minimum oxygen concentration). Equation 14 shows that the minimum oxygen value *c*_min_ decreases exponentially as the lumen length *L* increases, or the lumen velocity *U* decreases. This means that for a lower *c*_min_ requirement, a smaller flow velocity and longer fiber can be used.

Next it is assumed that the ratio *U*/*L* is prescribed so that Pe^*^ is given. Now the operating equation for *c*_min_ in terms of the ECS depth is:

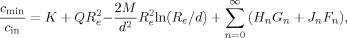
17 where

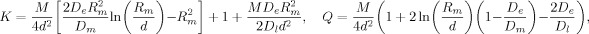
18

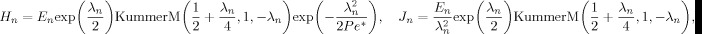
19 are all fixed constants. Given the values of these constants, 17 depends on *R*_*e*_ through the explicit appearance of *R*_*e*_ in 17 as well as *G*_*n*_ and *F*_*n*_ for 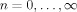. For a given value of *c*_min_, 17 can therefore be solved numerically to determine *R*_*e*_.


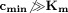
: In this scenario the numerical approach will be used, as outlined in Numerical Approach Section.

### Cell Types and Parameter Values

The parameters that will be kept fixed in our investigation are:

1. The oxygen diffusivities *D*_*l*_ = 3 × 10^−9^ m^2^ s^−1^, *D*_*m*_ = 3 × 10^−10^ m^2^ s^−1^, and *D*_*e*_ = 6 × 10^−9^ m^2^ s^−1^ (Ye et al., 41).
2. The lumen radius *R*_*l*_ = *d* = 100 µm and the depth of the membrane *s* = 20 µm (so that *R*_*m*_ = 120 µm) (Ye et al., 41).
3. The inlet oxygen concentration will be fixed for each individual cell type.

The kinetic data (i.e., *V*_max_ and *K*_*m*_) for a range of cell types, sourced from combined modeling and experimental studies in the literature, are shown in Table I. For cardiomyocytes, hepatocytes, and pancreatic cells we fix *c*_in_ = 0.22 mol m^−3^ (as is standard for culture medium Piret and Cooney, 29). However, chondrogenic differentiation is limited when the oxygen concentration exceeds approximately 0.1 mol m^−3^ (Lund-Olesen, 20; Treuhaft and McCarty, 38); therefore *c*_in_ = 0.1 mol m^−3^ is used for chondrocytes.

**Table I:** Oxygen uptake and culturing data for a range of cell types.

Cell type	*V*_max_ (mol m^−3^ s^−1^)	*K*_*m*_ (mol m^−3^)	Cell density (cells m^−3^)	*c*_min_ (mol m^−3^)	*c*_in_ (mol m^−3^)	Source
Neonatal rat cardiomyocytes	2.64 × 10^−3^	6.9 × 10^−3^	10^12^	8 × 10^−2^	0.22	Radisic et al. (30)
				6 × 10^−3^		Carrier et al. (5)
Primary rat hepatocytes	1.76 × 10^−3^	6.24 × 10^−3^	1.25 × 10^13^	2.1 × 10^−2^	0.22	Sullivan et al. (35)
						Consolo et al. (7)
Pancreatic βTC3 cells	6.37 × 10^−3^	1.0 × 10^−2^	2.8 × 10^14^	1.46 × 10^−2^	0.22	Tziampazis and Sambanis (39)
						Stabler et al. (34)
Bovine chondrocytes	4.8 × 10^−5^	5.0 × 10^−3^	1.4 × 10^14^	1 × 10^−2^	0.1	Malda et al. (21)
				1.32 × 10^−2^		Obradovic et al. (25, 26)
				2.2 × 10^−3^		Fermor et al. (11)

For a description of the various minimum oxygen concentrations, please refer to the main text. The *V*_max_ value for neonatal rat cardiomyocytes and primary rat hepatocytes have been multiplied by a cell volume fraction of 0.3, as per the modeling in Sullivan et al. (35). For the pancreatic cells it has also been assumed that each cell has a 10 µm diameter.

Note: For neonatal rat cardiomyocytes two values are listed. It has been observed that cardiac constructs cultivated in perfusion at oxygen concentrations of ∼80 µM exhibit weaker presence of cardiac markers and poorer organization of contractile apparatus compared with constructs cultivated at oxygen concentrations of ∼200 µM Carrier et al. (5); this explains the first value. The second value (6 µM) is a typical hypoxia value (Radisic et al., 30). The *c*_min_ value for primary rat hepatocytes is based the critical threshold value of 10 mmHg quoted in the literature Consolo et al. (7) (and transferred from a partial pressure into a concentration using Henry's law with an oxygen solubility value of 2.08 mmol m^−3^ mmHg). For pancreatic βTC3 cells, published experiments found that oxygen tensions above 7 mmHg were required for the cells to retain their secretory capacity Stabler et al. (34); using Henry's law gives the value in Table I. Finally, a range of minimum oxygen concentrations are presented for articular cartilage in the literature. In Obradovic et al. (25), it is hypothesized that articular cartilage is exposed to a minimum oxygen concentration in the range 0.01 mol m^−3^ to 0.08 mol m^−3^ in vivo, where lower oxygen concentrations are not detrimental to chondrocyte viability but can impact synthesis of extracellular matrix; this explains the first *c*_min_ value in Table I. In Fermor et al. (11), it is reported that the superficial zone of articular cartilage exists at above approximately 6% oxygen concentration, whereas the deep zone exists at <1%; this explains the final two *c*_min_ values of Table I.

### Validation of Analytical and Numerical Approaches

**Figure 2:**
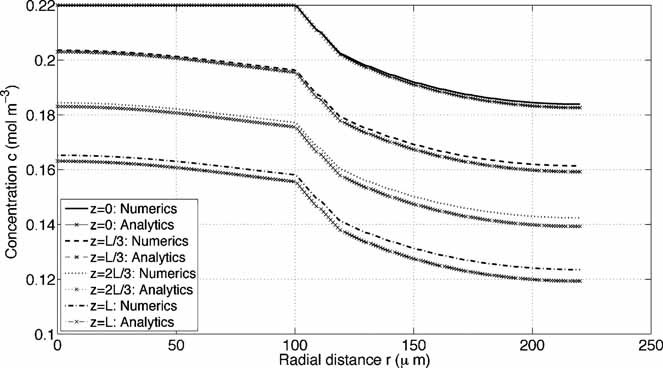
Comparison of the analytical and numerical approaches. The graph shows the radial oxygen concentration profiles for primary rat hepatocytes (see Table I) at fixed values of *z*, using both the analytical and numerical techniques. The fixed parameters are *U* = 1 × 10^−2^ ms^−1^, *L* = 10 cm, and *R_e_* = 220 µm.

The analytical approach is a reduction of the full model given by (2)–(6) and therefore should be validated. This validation could be performed against experimental data; however, this data is difficult to collect accurately and is not presented in sufficient detail in the literature. Given that the numerical approach is valid for all concentration values and solves the full model (2)–(6), it is appropriate to validate results of the analytical model against numerical solutions. This comparison is shown in Figure 2, where radial oxygen concentration profiles are shown (at fixed values *z* = 0, *L*/3, 2*L*/3, *L*) for the primary rat hepatocyte data in Table I (Sullivan et al., 35) when *U* = 1 × 10^−2^ m/s, *L* = 10 cm, *R*_*e*_ = 220 µm. For the analytical solutions, all sums have been truncated at 50 terms, that is, *n* = 49, for ease of computation. The agreement between the analytical and numerical results is very strong, although it becomes weaker as the concentrations decrease. The lowest concentration value is at the ECS outlet (when *r* = *R*_*e*_ and *z* = *L*); here both the analytical and numerical concentration values are 0.12 mol m^−3^ to two decimal places, with a percentage difference of 3.39% (which is within experimental error).

### Analytical and Numerical Results

It must first be decided whether to use the analytical or numerical strategy to provide operating data. Table II provides a summary of this decision making process. Data on *c*_min_ and *K*_*m*_ are provided for each cell type, together with the value of the ratio *c*_min_/*K*_*m*_. The analytical model is valid when *c*_min_ ≫ *K*_*m*_; here we choose a value of the ratio *c*_min_/*K*_*m*_ = 2 as the critical value so that if *c*_min_/*K*_*m*_ > 2 the analytical model is used, whereas if *c*_min_/*K*_*m*_ > 2 the numerical approach is used. Different critical values of *c*_min_/*K*_*m*_ could certainly be implemented, even on a cell-specific basis. The errors associated with using *c*_min_/*K*_*m*_ = 2 as the critical value are within the bounds of experimental error, and the errors associated with other modeling assumptions (e.g., the Krogh cylinder approximation). On this basis, the analytical model is appropriate for the cardiomyocytes (*c*_min_ = 8 × 10^−2^ mol m^−3^), hepatocytes, and chondrocytes (*c*_min_ = 1.32 × 10^−2^ mol m^−3^), whereas the numerical model is used for the remaining examples in Table II. We present data for the extreme cases of high and low oxygen requirements, that is, cardiomyocytes and chondrocytes, respectively.

**Table II:** Use of the analytical or numerical models. If *c*_min_/*K*_*m*_ > 2, the analytical model is used; otherwise the numerical model is used.

Cell type	*c*_min_/*c*_in_	*K*_*m*_/*c*_in_	*c*_min_/*K*_*m*_	Analytical model	Numerical model
Neonatal rat cardiomyocytes	0.36	0.031	11.6	✓	×
	0.027	0.031	0.87	×	✓
Primary rat hepatocytes	0.095	0.028	3.4	✓	×
Pancreatic βTC3 cells	0.066	0.045	1.5	×	✓
Bovine chondrocytes	0.1	0.05	2.0	×	✓
	0.13	0.05	2.64	✓	×
	2.2 × 10^−2^	0.05	0.44	×	✓

**Figure 3:**
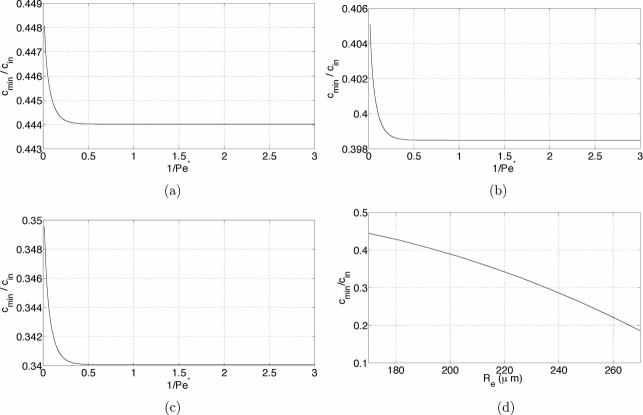
Operating equation data for the neonatal rat cardiomyocytes (analytical model). a–c: The dependence of *c*_min_/*c*_in_ on 1/Pe^*^ when *R_e_* is fixed. d: The dependence of *c*_min_/*c*_in_ on *R_e_* when Pe^*^ is fixed. a: [*R_e_* = 170 µm], (b) [*R_e_* = 195 µm], (c) [*R_e_* = 220 µm], and (d) Pe^*^ = 1/3.

**Figure 5:**
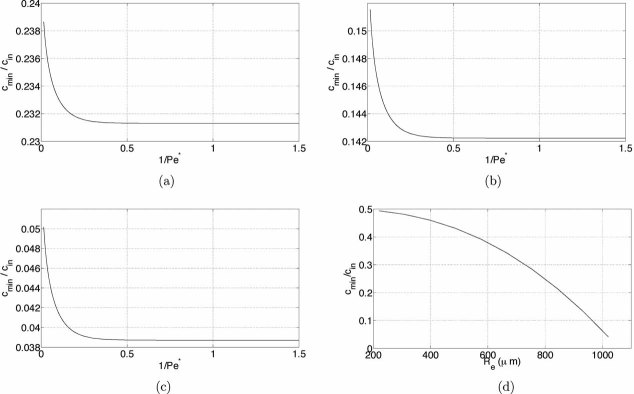
Operating equation data for the bovine chondrocytes (analytical model). a–c: The dependence of *c*_min_/*c*_in_ on 1/Pe^*^ when *R_e_* is fixed. d: The dependence of *c*_min_/*c*_in_ on *R_e_* when Pe^*^ is fixed. a: [*R_e_* = 820 µm], (b) [*R_e_* = 920 µm], (c) [*R_e_* = 1,020 µm], and (d) Pe^*^ = 1/3.

Figures 3a–c and 5a–c show the variation in *c*_min_/*c*_in_ (with *c*_in_ fixed) as a function of 1/Pe^*^ for fixed *R*_*e*_, as described by operating equation 14, for the cardiomyocytes and chondrocytes, respectively. As would be anticipated, *c*_min_ is largest for low values of 1/Pe^*^, corresponding to either a large lumen velocity *U* or shorter lumen length *L* (a larger *U* ensures increased delivery of oxygen to the cells through advection, whereas a shorter lumen length decreases the distance of the furthermost cells from the oxygen source). For each value of *R*_*e*_ the maximum variation in *c*_min_/*c*_in_ is of size 10^−2^, indicating that *c*_min_ is only weakly sensitive to the value of Pe^*^. For Pe^*^ < 2 (i.e., 1/Pe^*^ > 0.5) *c*_min_ is virtually constant, indicating a linear relationship between the values of *U* and *L* required to achieve a chosen value of *c*_min_.

**Figure 6:**
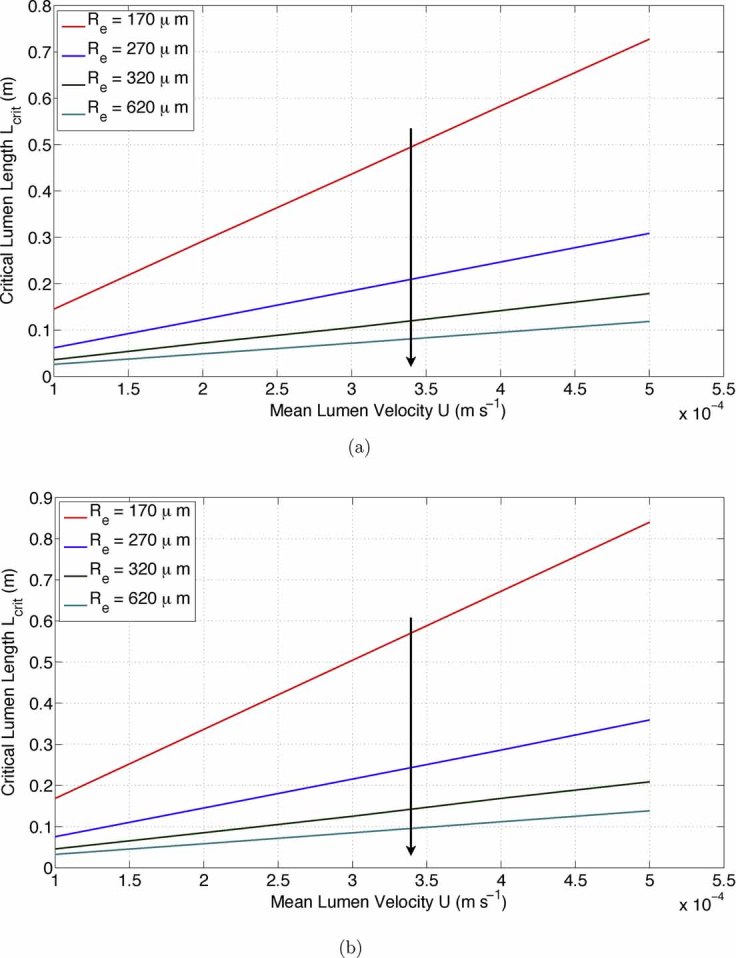
Numerical results for the bovine chondrocytes that show the relationship between *L*_crit_ and *U* for two different minimum oxygen requirements (arrows in direction of *R_e_* decreasing). a: [*c*_min_ = 1 × 10^−2^ mol m^−3^ and *c*_min_/*K_m_* = 2.0 held fixed], (b) [*c*_min_ = 2.2 × 10^−3^ mol m^−3^ and *c*_min_/*K_m_* = 0.44 held fixed].

Given that this linear relationship is representative of the low *c*_min_ regime, it is mimicked by the numerical results of Figures 4 and 6. These figures show how the critical lumen length, *L*_crit_ say, required to satisfy the minimum oxygen concentrations of Table I varies as a function of the lumen velocity *U*. For each cell type four different values of *R*_*e*_ were tested, each of which demonstrates a linear relationship between *L*_crit_ and *U* (with correlation factor 0.99). These figures can be used to read off a required *L*_crit_ and *U* value to satisfy the minimum oxygen requirements summarized in Table I. For example, for the cardiomyocytes with *R*_*e*_ = 270 µm with a lumen length of 10 cm, a lumen flow velocity of *U* ≍ 9 × 10^−3^ m s^−1^ will ensure *c* > 6 × 10^−3^ mol m^−3^ throughout the module.

**4:**
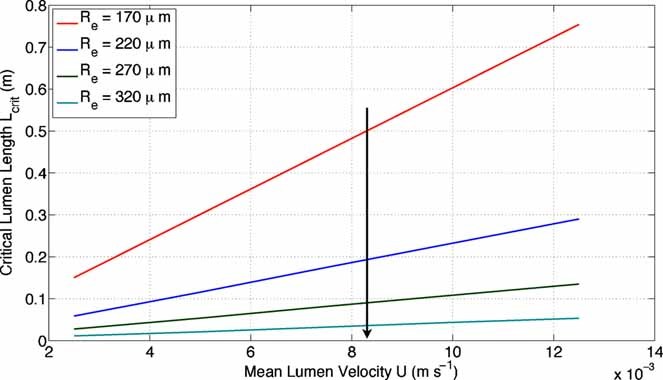
Numerical results for the neonatal rat cardiomyocytes that show the relationship between *L*_crit_ and *U* when *c*_min_ = 6 × 10^−3^ mol m^−3^ and *c*_min_/*K_m_* = 0.87 are held fixed (arrow in direction of *R_e_* decreasing).

In contrast, Figures 3d and 5d show the variation in *c*_min_/*c*_in_ (with *c*_in_ fixed in each case) as a function of *R*_*e*_ for fixed Pe^*^, as described by operating equation 17, for the cardiomyocytes and chondrocytes, respectively. As anticipated, *c*_min_ decreases as the ECS depth (i.e., *R*_*e*_) increases. The rate of this decay is heavily dependent on the uptake rate of oxygen by the cell population, *V*_max_, which is largest for the cardiomyocytes, and lowest for the chondrocytes. For example, a value of *c*_min_/*c*_in_ = 0.2 is sustained by the cardiomyocytes, hepatocytes, and chondrocytes when *R*_*e*_ ≍ 267 and 720 µm, respectively.

#### Case Study: Cells With a High Oxygen Requirement

Consider a HFB for culturing cardiomyocytes with the following known parameters: *c*_min_ = 8 × 10^−2^ mol m^−3^, *c*_in_ = 0.22 mol m^−3^, and *c*_min_/*K*_*m*_ = 11.6, together with the ECS depth fixed at 95 µm so that *R*_*e*_ = 215 µm. For HFB operation it is necessary to specify the inlet flowrate and fiber length to maintain the oxygen concentration above this minimum. Since *c*_min_/*K*_*m*_ > 2 and the ECS depth is fixed, the analytical approach (operating equation 14) will be used to determine the value of Pe^*^ (and corresponding possible values of *L* and *U*) that achieves *c*_min_/*c*_in_ = 0.36. For this scenario, *A* = 0.80 and the values of *B*_*n*_ and *C*_*n*_ for 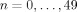 are given in the Supplementary Material C. Solving operating equation 14 yields Pe^*^ = 0.2 so that the ratio *U*/*L* = 6 × 10^−2^. Any values of *U* and *L* that satisfy this ratio will ensure *c* > 8 × 10^−2^ mol m^−3^ throughout the construct; two example values are *L* = 0.1 m and *U* = 6 × 10^−3^ m s^−1^.

By comparison, suppose the lumen flow velocity is fixed at *U* = 1 × 10^−2^ m s^−1^ and the lumen length at *L* = 0.1 m so that Pe^*^ = 1/3. Then operating equation 17 can be used to determine the ECS depth that achieves *c*_min_/*c*_in_ = 0.36. For this scenario, *K* = 1.01 and *Q* = 1.18 × 10^6^ m^−2^, and the values of *H*_*n*_ and *J*_*n*_ for 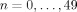 are given in the Supplementary Material C. Solving operating equation 17 with *c*_min_/*c*_in_ = 0.36 now gives *R*_*e*_ = 212.8 µm so that the ECS depth is 92.8 µm.

#### Case Study: Cells With a Low Oxygen Requirement

Next consider a HFB for culturing chondrocytes with the following known parameters: *c*_min_ = 2.2 × 10^−3^ mol m^−3^, *c*_in_ = 0.1 mol m^−3^, and *c*_min_/*K*_*m*_ = 0.44, together with the ECS depth fixed at 150 µm so that *R*_*e*_ = 270 µm. It is necessary to specify the inlet flowrate and fiber length to maintain the oxygen concentration above this minimum. Since *c*_min_/*K*_*m*_ ≤ 2 and the ECS depth is fixed, the numerical approach will be used to determine possible values of *L* and *U* that achieve *c*_min_/*c*_in_ = 2.2 × 10^−2^. For this scenario, we refer to Figure 6. Any values of *L* and *U* that lie on the blue line (*R*_*e*_ = 270 µm) are appropriate: an example is *U* = 2.8 × 10^−4^ m s^−1^ and *L* = 0.2 m.

By comparison, suppose the lumen flow velocity is fixed at *U* = 3 × 10^−4^ m s^−1^ and lumen length *L* = 0.5. The red line of Figure 6 dictates that *R*_*e*_ = 170 µm should be imposed in this case.

### Discussion

The strategy that has been outlined enables mathematical modeling techniques to inform bioreactor design based on the oxygen requirements of the cell type. Two different modeling approaches were employed to provide design and operating data that ensure the oxygen concentration throughout a HFB is held above a prescribed tissue-specific minimum value, *c*_min_ that ensures the growth of a functional cell population. When *c*_min_ ≫ *K*_*m*_ (the half-maximal oxygen concentration), oxygen uptake by the cell population was captured using zero-order kinetics, and operating equations were derived analytically. These operating equations provide insight into the relationship between the minimum oxygen concentration and the geometrical properties of the bioreactor, together with the operational parameters (such as inlet oxygen concentration and flow rate) than can be controlled by the user. A case study was presented that demonstrated how to use these operating equations for cell types with a high oxygen requirement. However, an analytical approach is not valid when 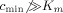. In this case, full Michaelis–Menten kinetics must be solved in the ECS using a numerical approach. This was achieved using the finite elements package “COMSOL Multiphysics,” and operating data on the relationship between lumen length and flow rate required to achieve a specific minimum oxygen concentration value were presented. This approach has the advantage of being valid for all concentration values; however, the full system of equations must be solved iteratively each time and this is a computationally intensive process. A case study was presented that demonstrated how to use these operating equations for cell types with a low oxygen requirement.

Previous studies into the modeling of tissue engineering bioreactors have focused on either numerical or analytical approaches (under various simplifying assumptions) in isolation. For example, Abdullah and Das (1), Chen and Palmer (6), Das (8), Pillarella and Zydney (28), Sullivan et al. (35, 36), and Ye et al. (41) employed various numerical techniques to solve full Michaelis–Menten kinetics for individual cell types in HFBs. By comparison, analytical approaches such as Piret and Cooney (29), Jayaraman (15), and Kim and Cooney (17) have been used to approximate Michaelis–Menten by zero- or first-order kinetics. However, zero-order kinetics have not previously been used to determine operating equations, whilst first-order kinetics are only valid when the substrate concentration is smaller than the half-maximal substrate concentration, *K*_*m*_. This is not appropriate in the development of oxygen-based operating equations for the use of HFB for tissue engineering, where the oxygen concentration must typically be maintained above *K*_*m*_ to ensure the growth of functional tissue. While these are all valid and workable models, they have not previously been integrated to provide a strategy that could be applied to any cell type to stipulate bioreactor design and operation.

## Conclusion

A strategy has been developed for modeling oxygen kinetics in tissue engineering HFB. The strategy allows operating parameters to be specified that ensure the oxygen concentration is maintained above a prescribed minimum throughout the HFB. The strategy dictates that the appropriate approach is based on whether the Michaelis–Menten kinetics can be reduced to zero-order; in the case of high oxygen requirements zero-order kinetics is appropriate and so the analytical approach is used. In the case of low oxygen requirements it is necessary to use full Michaelis–Menten kinetics and so a numerical approach is required. As such, the strategy developed here can be used for any cell type to specify operating parameters.

## Footnotes

1 Developed and distributed by COMSOL, Inc. Full details available online at http://www.comsol.com/.

## Nomenclature

*d*: radius of the lumen (m)
*s*: depth of the lumen wall (m)
*l*: depth of the ECS (m)
*L*: length of a single module (m)
*L*_crit_: critical length required to satisfy a minimum oxygen requirement (m)
*z*: axial length coordinate down the lumen
*r*: radial coordinate
**u**: fluid velocity vector (m s^−1^)
*U*: Mean velocity in the lumen (m s^−1^)
**e**_*z*_: unit vector in the *z*-direction
*c*: oxygen concentration (mol m^−3^)
**J**: oxygen flux (mol m^−2^ s^−1^)
*U*: velocity scale (m s^−1^)
*D*_*l*_: oxygen diffusion coefficient in the lumen (m^2^ s^−1^)
*D*_*w*_: oxygen diffusion coefficient in the wall (m^2^ s^−1^)
*D*_*e*_: oxygen diffusion coefficient in the ECS (m^2^ s^−1^)
*R*: uptake rate of oxygen (mol m^−3^ s^−1^)
*V*_max_: Maximal oxygen consumption rate (mol m^−3^ s^−1^)
*K*_*m*_: half-maximal oxygen concentration (mol m^−3^)
**n**: unit outward pointing normal to a surface
*c*_in_: fixed oxygen concentration at the lumen inlet (mol m^−3^)
*c*_min_: minimum oxygen concentration in the HFB (mol m^−3^)
*ε*: aspect ratio of the lumen
*Pe*: axial Péclet number in the lumen
*Pe*^*^: reduced Péclet number in the lumen
*R*_*l*_: outer lumen radius (m)
*R*_*m*_: outer membrane radius (m)
*R*_*e*_: outer ECS radius (m)
*M*: dimensionless constant that represents the balance of oxygen consumption versus diffusion in the ECS
γ: algebraically convenient parameter that depends on *R*_*e*_
*λ*_*n*_: eigenvalues of Sturm–Liouville problem for
*E*_*n*_: normalization constants of Sturm–Liouville problem for
*F*_*n*_, *G*_*n*_: Sturm–Liouville expansion constants for
*B*(*z*): dimensionless oxygen concentration on the lumen wall
*A*, *B*_*n*_, *C*_*n*_, *K*, *Q*, *H*_*n*_, *J*_*n*_: constants associated with the analytic operating equations
