# Supplementary material for: A strategy to determine operating parameters in tissue engineering hollow fiber bioreactors
Source: Biotechnol Bioeng. 2011 Jun;108(6):1450–61. doi: 10.1002/bit.23062 (PMC3579239; doi:10.1002/bit.23062)
Supplement: Supplementary file 1 [file bit0108-1450-sd1.pdf]

A Strategy To Determine Operating Parameters in Tissue Engineering Hollow Fibre Bioreactors

Supplementary Material

R.J. Shipley[1] , A.J. Davidson[3], K. Chan[2], J.B. Chaudhuri[3], S.L Waters[1] , M.J. Ellis[3⋆]

1: Oxford Centre for Industrial and Applied Mathematics, Mathematical Institute, 24–29 St. Giles', Oxford OX1 3LB.

2: Centre for Mathematical Biology, Mathematical Institute, 24–29 St. Giles', Oxford OX1 3LB.

3: Centre for Regenerative Medicine, Department of Chemical Engineering, University of Bath, Bath BA2 7AY.

Tel: 01225 384484

Fax: 01225 385713

Email: M.J.Ellis@bath.ac.uk

⋆: Corresponding author

A Analytical Model Reduction

The full modelling equations throughout the module are given by

$$\frac{\partial c_l}{\partial t} + \boldsymbol{\nabla}\cdot(\mathbf{u}c_l) = D_l \nabla^2 c_l \text{ in the lumen}, \quad \frac{\partial c_m}{\partial t} = D_m \nabla^2 c_m \text{ in the membrane}, \quad \frac{\partial c_e}{\partial t} = D_e \nabla^2 c_e - R(c_e) \text{ in the ECS} \quad (1)$$

where the reaction term is given by

$$R(c_e) = \frac{V_{max} c_e}{c_e + K_m}. \quad (2)$$

On the lumen/membrane and membrane/ECS boundaries we prescribe continuity of concentration and flux, so that

$$c_l = c_w \quad \text{and} \quad D_l \boldsymbol{\nabla} c_l \cdot \mathbf{n} = D_w \boldsymbol{\nabla} c_w \cdot \mathbf{n} \quad \text{on the lumen/membrane boundary}, \quad (3)$$

$$c_w = c_e \quad \text{and} \quad D_w \boldsymbol{\nabla} c_w \cdot \mathbf{n} = D_e \boldsymbol{\nabla} c_e \cdot \mathbf{n} \quad \text{on the membrane/ECS boundary}, \quad (4)$$

where $\mathbf{n}$ is the unit outward pointing normal to the relevant surface. Finally, we impose a prescribed concentration c_{in} at the lumen inlet, and no diffusive flux of concentration out of the outer ECS boundary,

$$c_l = c_{in} \quad \text{on } z = 0, \quad \text{and} \quad D_e \boldsymbol{\nabla} c_e \cdot \mathbf{n} = 0 \quad \text{on the outer ECS boundary}. \quad (5)$$

We mathematically reduce the full system of equations given by equations (1)–(5) to determine analytical expressions for the oxygen concentrations in each of the lumen, membrane and ECS.

First of all, we assume that the fibre is positioned symmetrically in the extra-lumen space and move to a radially symmetric setup described in cylindrical polar coordinates (defined by $x = r\cos\theta$, $y = r\sin\theta$ and $z = z$), neglecting θ–dependence so that $c = c(r, z)$.

We non-dimensionalise the system above to reduce the number of parameters and to estimate the relative importance of the various terms. We set

$$r = d\hat{r}, \quad z = L\hat{z}, \quad \mathbf{u}_l = U\hat{\mathbf{u}}, \quad t = \frac{L}{U}\hat{t}, \quad c = c_{in}\hat{c}, \quad (6)$$

where d is the lumen radius, L is the length of the lumen, U is the mean velocity in the lumen and c_{in} is the lumen inlet oxygen concentration. From here onwards we drop the 'hats' on the dimensionless variables. The system now becomes

$$\overline{\mathrm{Pe}}\left(\frac{\partial c_l}{\partial t} + 2\left(1 - r^2\right)\frac{\partial c_l}{\partial z}\right) = \frac{1}{r}\frac{\partial}{\partial r}\left(r\frac{\partial c_l}{\partial r}\right) + \epsilon^2 \frac{\partial c_l}{\partial z} \quad \text{in} \quad 0 < r < 1, \quad (7)$$

$$\frac{\overline{\mathrm{Pe}} D_l}{D_w}\frac{\partial c_w}{\partial t} = \frac{1}{r}\frac{\partial}{\partial r}\left(r\frac{\partial c_w}{\partial r}\right) + \epsilon^2 \frac{\partial^2 c_w}{\partial z^2} \quad \text{in} \quad 1 < r < R_w/d, \quad (8)$$

$$\frac{\overline{\mathrm{Pe}} D_l}{D_e}\frac{\partial c_e}{\partial t} = \frac{1}{r}\frac{\partial}{\partial r}\left(r\frac{\partial c_e}{\partial r}\right) + \epsilon^2 \frac{\partial^2 c_e}{\partial z^2} - M \quad \text{in} \quad R_w/d < r < R_e/d, \quad (9)$$

with boundary conditions

$$c_l = c_w \quad \text{and} \quad \frac{\partial c_l}{\partial r} = \frac{D_w}{D_l}\frac{\partial c_w}{\partial r} \quad \text{on } r = 1, \tag{10}$$

$$c_w = c_e \quad \text{and} \quad \frac{\partial c_w}{\partial r} = \frac{D_e}{D_w}\frac{\partial c_e}{\partial r} \quad \text{on } r = R_w/d, \tag{11}$$

$$\frac{\partial c_e}{\partial r} = 0 \quad \text{on } r = R_e/d, \tag{12}$$

$$c_l\,(r,0) = 1, \tag{13}$$

where all parameters are defined in the main paper.

A.1 Solution in the Membrane and ECS

Next we solve the system described by equations (7)–(13) in steady state to predict the oxygen distribution throughout the module. We integrate the steady-state versions of equations (8) and (9) for the concentration in the wall and ECS to give

$$c_w = A\,(z)\ln r + B\,(z)\,, \quad c_e = \frac{Mr^2}{4} + C\,(z)\ln r + D\,(z)\,, \tag{14}$$

where $A\,(z)$, $B\,(z)$, $C\,(z)$ and $D\,(z)$ are arbitrary functions of z. Inputting the no-flux boundary condition out of the ECS outlet (from equation (12)), together with the continuity of concentration and flux boundary conditions on $r = R_w/d$ (equation (11)) yields

$$c_m = \frac{MD_e}{2D_m d^2}\left(R_m^2 - R_e^2\right)\ln r + B\,(z)\,, \tag{15}$$

$$c_e = \frac{M}{4d^2}\left[(dr)^2 - R_m^2 + \frac{2D_e}{D_m}\left(R_m^2 - R_e^2\right)\ln\left(\frac{R_m}{d}\right) + 2R_e^2\ln\left(\frac{R_m}{dr}\right)\right] + B\,(z)\,, \tag{16}$$

where $B\,(z)$ remains unknown. To determine B, and the solution in the lumen, we must now solve equation (7) (in steady-state), subject to the boundary conditions

$$\frac{\partial c_l}{\partial r}\,(1,z) = \gamma, \quad c_l\,(r,0) = 1, \tag{17}$$

where γ is defined by

$$\gamma = \frac{MD_e}{2D_l d^2}\left[R_m^2 - R_e^2\right]\,, \tag{18}$$

and we also require that $\frac{\partial c_l}{\partial r}$ is bounded at $r = 0$.

A.2 Solution in the Lumen

We perform a change of variables to give a homogeneous derivative boundary condition on $r = 1$. We let

$$c_l = c + \gamma r + 1, \tag{19}$$

upon which the system in the lumen becomes

$$2\overline{\mathrm{Pe}}_r \frac{\partial c}{\partial z} = \frac{1}{r\left(1-r^2\right)} \frac{\partial}{\partial r}\left(r\frac{\partial c}{\partial r}\right) + \frac{\gamma}{r\left(1-r^2\right)} \quad \text{in } 0 < r < 1, \tag{20}$$

$$\frac{\partial c}{\partial r}\left(1, z\right) = 0, \tag{21}$$

$$\frac{\partial c}{\partial r} \text{ finite as } r \to 0, \tag{22}$$

$$c\left(r, 0\right) = -\gamma r. \tag{23}$$

A.2.1 Solution of the Homogeneous Counterpart to (20)–(22)

First of all, we consider the homogeneous counterpart to (20) given by

$$2\overline{\mathrm{Pe}}_r \frac{\partial c}{\partial z} = \frac{1}{r\left(1-r^2\right)} \frac{\partial}{\partial r}\left(r\frac{\partial c}{\partial r}\right) \quad \text{in } 0 < r < 1\,, \tag{24}$$

and solve via separation of variables by seeking a solution of the form $c\left(r, z\right) = f\left(r\right) g\left(z\right)$. This yields

$$\frac{1}{r\left(1-r^2\right)} \frac{1}{f} \frac{\mathrm{d}}{\mathrm{d}r}\left(r\frac{\mathrm{d}f}{\mathrm{d}r}\right) = \frac{2\overline{\mathrm{Pe}}_r}{g}\frac{\mathrm{d}g}{\mathrm{d}z}. \tag{25}$$

The LHS of equation (25) is dependent on r only, whilst the RHS is dependent on z only. Therefore both sides must constant, and indeed this constant must be negative (the concentration must decay as z increases from the inlet at $z = 0$). Defining this constant to be $-\lambda^2$, the system for $f\left(r\right)$ becomes

$$\frac{\mathrm{d}}{\mathrm{d}r}\left(r\frac{\mathrm{d}f}{\mathrm{d}r}\right) + \lambda^2 r\left(1-r^2\right) f = 0 \quad \text{for } 0 < r < 1, \tag{26}$$

with boundary conditions

$$\frac{\mathrm{d}f}{\mathrm{d}r}\left(1\right) = 0 \quad \text{and} \quad \frac{\mathrm{d}f}{\mathrm{d}r} \text{ finite as } r \to 0. \tag{27}$$

This is a *Sturm-Liouville problem* – for further details please refer to Abramowitz & Stegun (1965). To solve for $f\left(r\right)$, we perform two transformations

$$X = \lambda r^2, \quad \Theta = \exp\left(\lambda r^2/2\right) f\left(r\right)\,, \tag{28}$$

which transform (26) to

$$X\frac{\mathrm{d}^2\Theta}{\mathrm{d}X^2}+(1-X)\frac{\mathrm{d}\Theta}{\mathrm{d}X}-\left(\frac{1}{2}-\frac{\lambda}{4}\right)\Theta=0, \tag{29}$$

which is known as the *Kummer equation.* The Kummer equation is normally written in the form

$$x\frac{\mathrm{d}^2y}{\mathrm{d}x^2}+(\nu-x)\frac{\mathrm{d}y}{\mathrm{d}x}-\mu y=0; \tag{30}$$

here we have $\nu=1$ and $\mu=\frac{1}{2}-\frac{\lambda}{4}$. The Kummer equation has two linearly independent solutions, denoted KummerM (μ,ν,x) and KummerU (μ,ν,x) (both hypergeometric functions), and so our solution here can be written as a linear sum of the two,

$$\Theta=E\,\mathrm{KummerM}\left(\frac{1}{2}-\frac{\lambda}{4},1,X\right)+F\,\mathrm{KummerU}\left(\frac{1}{2}-\frac{\lambda}{4},1,X\right), \tag{31}$$

where E and F are integration constants to be determined. A brief summary of the properties of the KummerM and KummerU functions required here is given below; for further details please refer to Abramowitz & Stegun (1965).

Important Properties of the KummerM and KummerU Functions

1. Both KummerM(μ,ν,x) and KummerU(μ,ν,x) are finite in the limit as $x\to 0$.
2. $\frac{\mathrm{d}}{\mathrm{d}x}\mathrm{KummerM}\left(\mu,\nu,x\right)=\frac{\mu}{\nu}\mathrm{KummerM}\left(\mu+1,\nu+1,x\right)$.
3. $\mathrm{KummerM}\left(\mu,\nu,x\right)=\exp\left(x\right)\mathrm{KummerM}\left(\nu-\mu,\nu,-x\right)$.

The requirement that $\mathrm{d}f/\mathrm{d}r$ is finite as $r\to 0$ yields $F=0$ (to avoid a $1/\sqrt{X}$ singularity as $X\to 0$) – this uses property 1. The boundary condition at $r=1$ from (27) provides the following *eigenvalue equation* for λ,

$$\left(1-\frac{\lambda}{2}\right)\mathrm{KummerM}\left(\frac{1}{2}+\frac{\lambda}{4},2,-\lambda\right)-\mathrm{KummerM}\left(\frac{1}{2}+\frac{\lambda}{4},1,-\lambda\right)=0. \tag{32}$$

The determination of this equation uses property 2. above to evaluate the derivative, and 3. to reduce oscillations in the eigenvalue equation. Equation (32) has infinitely many solutions for λ which may be determined by solving Equation (32) numerically; we denote them by λ_n, where $n=0\ldots\infty$. Each eigenvalue λ_n corresponds to a different solution for f (an *eigenfunction*), which we now denote by f_n. These functions f_n are given by

$$f_n=E_n\exp\left(\frac{\lambda_n r^2}{2}\right)\mathrm{KummerM}\left(\frac{1}{2}+\frac{\lambda_n}{4},1,-\lambda_n r^2\right), \tag{33}$$

where Sturm-Liouville theory dictates that the coefficients E_n may be fixed used the normalization condition

$$\int_{r=0}^{1}s\left(r\right)f_n^2\left(r\right)\,\mathrm{d}r=1, \tag{34}$$

for each $n = 0 \ldots \infty$, where $s(r) = r\left(1 - r^2\right)$ are the *weight functions* of the Sturm-Liouville equation (26). Therefore,

$$E_n^2 = 1/\int_{r=0}^{1} r\left(1-r^2\right)\exp\left(\lambda_n r^2\right)\left(\mathrm{KummerM}\left(\frac{1}{2}+\frac{\lambda_n}{4}, 1, -\lambda_n r^2\right)\right)^2 \mathrm{d}r. \tag{35}$$

A.2.2 Solution of the Non-Homogeneous Problem (20)–(22)

Now we return to the non-homogeneous problem given by (20)–(22), and pick up the non-homogeneous term $\gamma/r\left(1-r^2\right)$ using the radial solution, f. Sturm-Liouville theory states that this non-homogeneous term can be expressed as a linear sum of the eigenfunctions f_n, which act as basis functions (analogous to a Fourier series expansion). Therefore,

$$\frac{\gamma}{r\left(1-r^2\right)} = \sum_{n=0}^{\infty} F_n f_n(r), \tag{36}$$

where the F_n are constants given by

$$F_n = \int_{r=0}^{1} \frac{\gamma s(r)}{r\left(1-r^2\right)} f_n(r)\,\mathrm{d}r = \int_{r=0}^{1} \gamma f_n(r)\,\mathrm{d}r\,. \tag{37}$$

Next we express the solution for c as a linear sum over all the eigenvalues and eigenfunctions (admissible because the equations (20)–(23) are linear),

$$c = \sum_{n=0}^{\infty} f_n(r)\, g_n(z)\,, \tag{38}$$

where the $g_n(z)$ are undetermined. Substituting the two sum expressions (36) and (38) into the Equation (20) for c and using Equation (26) to simplify the derivative terms in f_n yields the following ordinary differential equation for each g_n $(n = 0 \ldots \infty)$

$$2\overline{\mathrm{Pe}}_r \frac{\mathrm{d}g_n}{\mathrm{d}z} + \lambda_n^2 g_n(z) = F_n. \tag{39}$$

The solution to (39) can be written in the form

$$g_n = G_n \exp\left(-\frac{\lambda_n^2}{2\overline{\mathrm{Pe}}_r} z\right) + \frac{F_n}{\lambda_n^2}, \tag{40}$$

where the integration constants G_n are undetermined. Indeed, substituting for c from Equation (38) into the remaining boundary condition (23) yields

$$\sum_{n=0}^{\infty} g_n(0)\, f_n(r) = -\gamma r. \tag{41}$$

Expressing $g_n(0)$ as a Sturm-Liouville sum yields

$$g_n(0) = \int_0^1 -\gamma r^2\left(1-r^2\right) f_n(r)\,\mathrm{d}r\,, \tag{42}$$

where the expression for g_n given by Equation (40) means that

$$g_n(0) = G_n + \frac{F_n}{\lambda_n^2}. \tag{43}$$

Therefore the constants G_n are given by

$$G_n = -\frac{F_n}{\lambda_n^2} + \int_0^1 -\gamma r^2 \left(1 - r^2\right) f_n(r) \, \mathrm{d}r. \tag{44}$$

The final solution for c_l is summarized in the main paper.

B Eigenvalues and Normalization Constants for the Sturm-Liouville Problem

The eigenvalues λ_n, and normalization constants E_n (for $n = 0, \ldots \infty$) as given by Equations (32) and (35) are independent of the geometry of the bioreactor and the cell population properties. The values of λ_n and E_n for $n = 0, \ldots 49$ are summarized in Table 2.

n	λ_n	E_n
0	5.06751	4.58666
1	9.15761	6.0934
2	13.1972	7.29187
3	17.2202	8.31844
4	21.2355	9.23112
5	25.2465	10.0611
6	29.2549	10.8275
7	33.2615	11.5431
8	37.2669	12.2168
9	41.2714	12.8552
10	45.2752	13.4633
11	49.2785	14.0451
12	53.2813	14.6037
13	57.2838	15.1417
14	61.286	15.6612
15	65.288	16.164
16	69.2898	16.6517
17	73.2914	17.1254
18	77.2929	17.5864
19	81.2943	18.0356

20	85.2955	18.4739
21	89.2967	18.902
22	93.2978	19.3206
23	97.2988	19.7304
24	101.3	20.1318
25	105.301	20.5254
26	109.301	20.9115
27	113.302	21.2907
28	117.303	21.6632
29	121.304	22.0294
30	125.304	22.3896
31	129.305	22.7441
32	133.305	23.0932
33	137.306	23.4371
34	141.306	23.776
35	145.307	24.1101
36	149.307	24.4397
37	153.308	24.7649
38	157.308	25.0859
39	161.309	25.4028
40	165.309	25.7158
41	169.31	26.025
42	173.31	26.3306
43	177.31	26.6327
44	181.311	26.9315
45	185.311	27.2269
46	189.311	27.5192
47	193.312	27.8084
48	197.312	28.0946
49	201.312	28.377

Table 1: The values of λ_n and E_n for $n = 0, \ldots 49$

C Worked Example of the Operating Equations: Cardiomyocytes

C.1 ECS Depth Fixed

n	B_n	C_n
0	0.0113993	-0.00237277
1	0.0051295	-0.000420139
2	0.00297741	-0.00014473
3	0.00195891	-6.78109×10^{-5}
4	0.00139676	-3.76759×10^{-5}
5	0.00105284	-2.33015×10^{-5}
6	0.000826219	-1.55143×10^{-5}
7	0.00066838	-1.09022×10^{-5}
8	0.000553649	-7.98395×10^{-6}
9	0.000467386	-6.04048×10^{-6}
10	0.000400727	-4.69235×10^{-6}
11	0.000348041	-3.72553×10^{-6}
12	0.0003056	-3.01269×10^{-6}
13	0.000270857	-2.47466×10^{-6}
14	0.000242016	-2.06033×10^{-6}
15	0.000217784	-1.73566×10^{-6}
16	0.000197207	-1.47736×10^{-6}
17	0.000179567	-1.26907×10^{-6}
18	0.000164319	-1.09911×10^{-6}
19	0.000151039	-9.58919×10^{-7}
20	0.000139394	-8.42176×10^{-7}
21	0.000129119	-7.44109×10^{-7}
22	0.000120003	-6.61076×10^{-7}
23	0.000111874	-5.90262×10^{-7}
24	0.00010459	-5.29469×10^{-7}
25	9.80348×10^{-5}	-4.7696×10^{-7}
26	9.21125×10^{-5}	-4.31349×10^{-7}
27	8.67419×10^{-5}	-3.91525×10^{-7}
28	8.18546×10^{-5}	-3.56584×10^{-7}
29	0.000077393	-3.25789×10^{-7}
30	7.33076×10^{-5}	-2.98534×10^{-7}
31	6.95563×10^{-5}	-2.74317×10^{-7}
32	6.61026×10^{-5}	-2.52721×10^{-7}
33	0.000062915	-2.33393×10^{-7}
34	5.99662×10^{-5}	-2.16041×10^{-7}
35	5.72322×10^{-5}	-2.00413×10^{-7}
36	5.46921×10^{-5}	-1.86298×10^{-7}
37	5.23275×10^{-5}	-1.73514×10^{-7}
38	5.01221×10^{-5}	-1.61905×10^{-7}
39	4.80617×10^{-5}	-1.51338×10^{-7}
40	4.61334×10^{-5}	-1.41696×10^{-7}

41	4.43258×10^{-5}	-1.32878×10^{-7}
42	4.26288×10^{-5}	-1.24797×10^{-7}
43	4.10333×10^{-5}	-1.17377×10^{-7}
44	3.95312×10^{-5}	-1.1055×10^{-7}
45	3.81151×10^{-5}	-1.04257×10^{-7}
46	3.67784×10^{-5}	-9.84458×10^{-8}
47	0.000035515	-9.30709×10^{-8}
48	3.43197×10^{-5}	-8.80911×10^{-8}
49	3.31873×10^{-5}	-8.34704×10^{-8}

Table 2: Worked example of the operating equations: cardiomyocytes. The values of B_n and C_n for $n = 0, \ldots 49$ for neonatal rat cardiomyocytes with $R_e = 215\,\mu$m

C.2 Lumen Flow Velocity and Length Fixed

n	H_n	J_n
0	-4.22×10^{-17}	-8.80×10^{-2}
1	5.64×10^{-55}	2.87×10^{-2}
2	-8.76×10^{-114}	-1.45×10^{-2}
3	1.74×10^{-193}	8.81×10^{-3}
4	-4.61×10^{-294}	-5.96×10^{-3}
5	0.00	4.32×10^{-3}
6	0.00	-3.29×10^{-3}
7	0.00	2.59×10^{-3}
8	0.00	-2.10×10^{-3}
9	0.00	1.74×10^{-3}
10	0.00	-1.46×10^{-3}
11	0.00	1.25×10^{-3}
12	0.00	-1.08×10^{-3}
13	0.00	9.47×10^{-4}
14	0.00	-8.36×10^{-4}
15	0.00	7.44×10^{-4}
16	0.00	-6.67×10^{-4}
17	0.00	6.01×10^{-4}
18	0.00	-5.45×10^{-4}
19	0.00	4.96×10^{-4}
20	0.00	-4.54×10^{-4}
21	0.00	4.18×10^{-4}
22	0.00	-3.85×10^{-4}
23	0.00	3.56×10^{-4}

24	0.00	-3.31×10^{-4}
25	0.00	3.08×10^{-4}
26	0.00	-2.88×10^{-4}
27	0.00	2.69×10^{-4}
28	0.00	-2.53×10^{-4}
29	0.00	2.37×10^{-4}
30	0.00	-2.24×10^{-4}
31	0.00	2.11×10^{-4}
32	0.00	-2.00×10^{-4}
33	0.00	1.89×10^{-4}
34	0.00	-1.79×10^{-4}
35	0.00	1.70×10^{-4}
36	0.00	-1.62×10^{-4}
37	0.00	1.54×10^{-4}
38	0.00	-1.47×10^{-4}
39	0.00	1.40×10^{-4}
40	0.00	-1.34×10^{-4}
41	0.00	1.28×10^{-4}
42	0.00	-1.23×10^{-4}
43	0.00	1.18×10^{-4}
44	0.00	-1.13×10^{-4}
45	0.00	1.09×10^{-4}
46	0.00	-1.05×10^{-4}
47	0.00	1.01×10^{-4}
48	0.00	-9.70×10^{-5}
49	0.00	9.34×10^{-5}

Table 3: Worked example of the operating equations: cardiomyocytes. The values of H_n and J_n for $n = 0, \dots 49$ for neonatal rat cardiomyocytes with $\overline{\mathrm{Pe}} = 1/3$

References

Abramowitz, M. & Stegun, I. (Eds.) (1965). *Handbook of Mathematical Functions*. Nat. Bur. Standards.
